# Transcription factor clusters as information transfer agents

**DOI:** 10.1126/sciadv.adp3251

**Published:** 2025-01-01

**Authors:** Rahul Munshi, Jia Ling, Sergey Ryabichko, Eric F. Wieschaus, Thomas Gregor

**Affiliations:** ^1^ Joseph Henry Laboratories of Physics, Princeton University, Princeton, NJ 08544, USA.; ^2^ Lewis-Sigler Institute for Integrative Genomics, Princeton University, Princeton, NJ 08544, USA.; ^3^ Department of Molecular Biology and Howard Hughes Medical Institute, Princeton University, Princeton, NJ 08544, USA.; ^4^ Department of Stem Cell and Developmental Biology, CNRS UMR3738 Paris Cité, Institut Pasteur, 25 rue du Docteur Roux, 75015 Paris, France.

## Abstract

Deciphering how genes interpret information from transcription factor (TF) concentrations within the cell nucleus remains a fundamental question in gene regulation. Recent advancements have revealed the heterogeneous distribution of TF molecules, posing challenges to precisely decoding concentration signals. Using high-resolution single-cell imaging of the fluorescently tagged TF Bicoid in living *Drosophila* embryos, we show that Bicoid accumulation in submicrometer clusters preserves the spatial information of the maternal Bicoid gradient. These clusters provide precise spatial cues through intensity, size, and frequency. We further discover that Bicoid target genes colocalize with these clusters in an enhancer-binding affinity-dependent manner. Our modeling suggests that clustering offers a faster sensing mechanism for global nuclear concentrations than freely diffusing TF molecules detected by simple enhancers.

## Teaser

Live imaging shows that regulatory protein clusters may aid fast and precise delivery of transcriptional information to gene loci.

## INTRODUCTION

Transcription factors (TFs) play a pivotal role in regulating gene expression by interacting with DNA regulatory elements known as enhancers (*1*–*3*). These enhancers often exhibit concentration-dependent behavior, activating or repressing gene expression only within specific TF concentration thresholds (*4*, *5*). The remarkable sensitivity of enhancers to subtle variations in the nuclear concentration of TF molecules implies that genes and enhancers carry out precise measurements of TF concentration (*6*, *7*).

However, the challenge arises because TF levels are often quite low and TF molecules are not uniformly distributed in the nucleus (*8*). Instead, they assemble into dynamic transcriptional microenvironments called transcriptional hubs (*9*–*11*). These TF molecule accumulations are believed to form through transient clustering mechanisms (*12*–*15*) or through liquid-liquid phase separations (LLPS) (*16*–*18*). Separation of LLPS clusters reflects saturation kinetics, such that increasing concentration of the minor component results in increased size of droplets rather than an increase in the concentration within droplets (*19*, *20*). Whether droplet size provides a useful proxy for global nuclear concentration is unclear.

Here, we aim to investigate whether the physical features of these TF assemblies such as size, concentration, or total molecular content accurately reflect the nuclear concentration. We leverage the unique characteristics of the *Drosophila* TF Bicoid (Bcd), known for its varying concentration along the anterior–posterior (AP) axis of the early embryo (*21*). Despite low nuclear concentrations, Bcd exhibits an extraordinarily reproducible profile, revealing precision in positional information comparable to the size of a single cell (*22*–*24*).

Various imaging approaches have unveiled that, similar to many other TFs, Bcd is not homogeneously distributed in the nucleus (*25*–*28*). Instead, it forms numerous cluster-like droplets enriched with chromatin accessibility factors like Zelda (*9*) and actively transcribed canonical Bcd target genes, such as *Hunchback* (*hb*), (*29*). The higher concentrations within Bcd accumulations are believed to enhance transcription by increasing the local concentration near target enhancers (*15*, *30*). However, for these clusters to be functionally relevant to Bcd’s well-characterized role in patterning, some features of the observed clusters must convey positional information with a precision similar to the nuclear concentration profile.

Here we developed a quantitative imaging strategy to decipher which features of Bcd accumulations maintain information about concentration. Contrary to simple LLPS models, we found that cluster size remains independent of concentration, while the cluster concentration varies linearly with nuclear Bcd concentration. These clusters localize at the locus of active target genes, precisely conferring information about cellular position along the AP axis in the embryo. We use these data to quantitatively explore the impact of clustering on information transfer and discuss the circumstances where clustering might be a preferred mechanism as opposed to the gene interacting with the TF molecules freely diffusing in the nucleus.

## RESULTS

### Heterogeneity of nuclear TF distribution

**Fig. 1.**
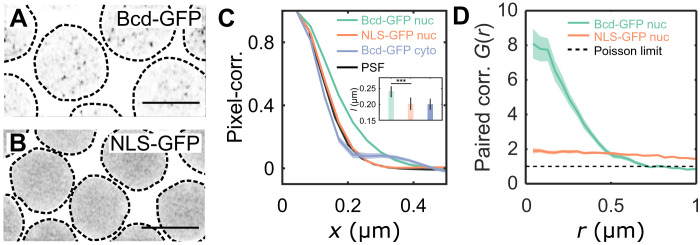
Quantitative characterization of nuclear Bcd heterogeneity. (**A** and **B**) Confocal (Zeiss-Airyscan) images of cross sections of Bcd-GFP (A) and NLS-GFP (B) expressing blastoderm nuclei in living *Drosophila* embryos (NC14). Scale bars, 5 μm. The broken lines represent a guide to the eye for nuclear boundaries. (**C**) Pixel correlations computed on the nuclear pixels in 2D nuclear cross-sectional images (Materials and Methods) expressing Bcd-GFP (green, 44 nuclei from 5 embryos) and NLS-GFP (orange, 27 nuclei from 3 embryos) and from pixels within the cytoplasm of Bcd-GFP expressing embryos (gray, 5 embryos). For comparison, the objective’s point-spread-function (PSF) is in black. Inset shows mean and SDs of the computed correlation lengths *l* for nucleoplasmic Bcd-GFP (*l* = 0.24 ± 0.02 μm), nucleoplasmic NLS-GFP (*l* = 0.20 ± 0.02 μm), and cytoplasmic Bcd-GFP (*l* = 0.20 ± 0.02 μm). (**D**) Radial distribution function [or pair-correlation function, *G*(*r*)] for the local maxima distribution expressed as a function of distance *r* from the center. *G*(*r*) was calculated on time-projected (60 frames each) local intensity maxima centroid maps (fig. S5 and Materials and Methods), averaged over multiple nuclei [same nuclei and embryo count as in (C)]. A distinct peak in *G*(*r*) indicates temporally persistent confinement of the local maxima, as seen for Bcd-GFP-expressing nuclei. For NLS-GFP, the continuous reduction in the radial function suggests a gradual decline in intensity near the nuclear edges without submicrometer accumulations. The dashed line $\left[G\left(r\right)=1\right]$ corresponds to a perfectly uniform distribution, the Poisson limit.

To gain quantitative insights into the heterogeneous distribution of Bcd within nuclei, we revisited its spatial organization. All data presented in this study were derived from live samples unless stated otherwise. Bcd molecules are distributed within the nucleus as both freely diffusing molecules and those interacting with chromatin (*21*, *31*). Cross-sectional images of nuclei expressing Bcd tagged with green fluorescent protein (Bcd-GFP) (1-μm-thick z-sections) showed multiple focal accumulations per cross section (Fig. 1A and figs. S1, A to D, and S2; see also fig. S3). In contrast, embryos expressing a fusion construct with a nuclear localization sequence and GFP (NLS-GFP), where molecules diffuse freely without chromatin interaction, exhibited a uniform distribution with no such focal accumulations (Fig. 1B).

Quantitative analysis of these focal accumulations, using pixel correlation functions, showed an average correlation length of 240 ± 20 nm for nuclear Bcd-GFP, compared to 200 ± 20 nm for NLS-GFP and cytoplasmic Bcd-GFP (Fig. 1C). The latter two coincide with the point spread function (PSF) of the microscope (fig. S1F), indicating that they are freely diffusing, whereas nuclear Bcd-GFP forms focal accumulations larger than the diffraction limit.

To examine the spatiotemporal persistence of these accumulations, we captured short videos (30 s) of nuclear cross sections and analyzed local GFP fluorescence intensity maxima in each frame (fig. S4, Materials and Methods). Projection maps of these maxima revealed that Bcd-GFP accumulations tend to cluster within specific confinement areas in the nucleus, contrasting with the more dispersed maxima observed in NLS-GFP nuclei (fig. S5, A, B, D, and E). Pair-correlation analysis (*32*) estimated an effective confinement radius of 370 ± 50 nm for Bcd-GFP (Fig. 1D), a pattern absent in NLS-GFP nuclei. The density of local maxima was approximately eight times higher within these confinement areas (fig. S5, F and G), suggesting persistent submicrometer clustering.

We further quantified cluster lifetime (τ_on_) and formation frequency (inverse of τ_off_) from maxima within confinement areas, yielding values of τ_on_ = 2.4 ± 0.3 s and τ_off_ = 1.6 ± 0.3 s, respectively (fig. S5H). The cluster detection probability, calculated as τ_on_/(τ_on_ + τ_off_), was approximately 58% (fig. S5I). These results indicate that Bcd forms persistent submicrometer clusters within the nucleus. To explore their role in information transfer to target genes, we next characterize the biophysical properties of these clusters.

### Quantitative properties of Bcd clusters

To characterize the three-dimensional (3D) extent and biophysical properties of Bcd clusters, we used a different approach from the maxima detection method described earlier. Given that any cluster must be at least the size of the PSF in the *x*-*y* plane (>4 pixels), we applied a 3-pixel cutoff to exclude spurious spots. In addition, based on the persistence data (fig. S5H), we determined that, with an imaging frame time of ∼500 ms, a cluster should span at least two consecutive z frames (Materials and Methods). This method enabled the identification of 40 to 70 clusters per nucleus.

**Fig. 2.**
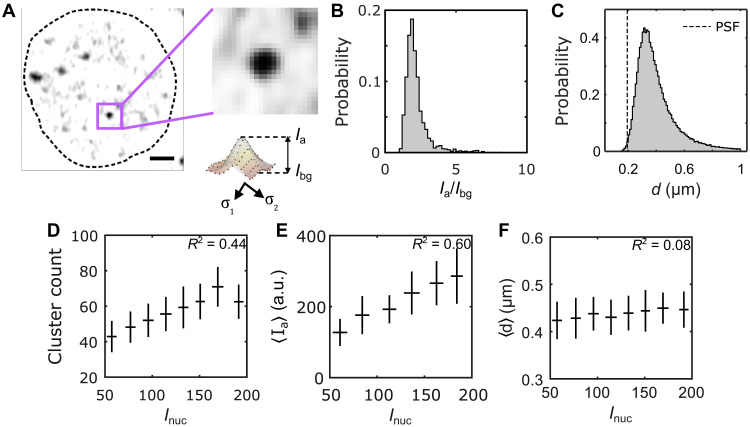
Biophysical properties of Bcd clusters. (**A**) A single nucleus showing Bcd-GFP heterogeneities. The close-up image (right) shows a single Bcd-GFP cluster. Cluster intensity fit with a 2D Gaussian (see profile below). The cluster amplitude *I*_a_, the cluster background intensity *I*_bg_, and the cluster size *d* are extracted from fit parameters (Materials and Methods). Scale bar, 1 μm. (**B**) A histogram of the signal-to-background ratio (*I*_a_/*I*_bg_) for 99,671 clusters from 2027 nuclei in 14 embryos expressing Bcd-GFP is plotted. (**C**) A histogram of the cluster size *d*, computed from the same clusters as in (B) is shown. The vertical dashed line representing the size of the PSF is included to compare with the size of the detected clusters. (**D** to **F**) The number of clusters per nucleus (D), the nuclear average of cluster amplitude 〈*I*_a_〉 (E), and the nuclear average of cluster size 〈*d*〉 (F) are plotted against nuclear Bcd-GFP intensity, *I*_nuc_. Error bars represent the mean ± SD for data in each *I*_nuc_ bin, calculated via bootstrap sampling of data within each bin. The coefficient of determination for each plot in (D), (E), and (F) is indicated in the respective panels.

Individual cluster properties were quantified using 2D Gaussian fits of the GFP intensity profile at the cluster centroid’s *z* plane (Fig. 2A). These fits provided an estimate for the effective cluster diameter *d*, with an average size of 〈*d*〉 = 400 ± 140 nm per nucleus (Fig. 2C, fig. S6, and Materials and Methods). The cluster size distribution lacked a left tail around the PSF limit, despite using a smaller cutoff (150 nm), suggesting that the detectable clusters are not diffraction limited under our imaging conditions. Subdiffraction clusters may exist with low intensities or high transience, making them undetectable in this study.

To quantify cluster concentration, we defined the parameters *I*_a_, the peak cluster intensity (cluster amplitude), and *I*_bg_, the Bcd-GFP intensity in the space surrounding the cluster (Fig. 2A, fig. S7, and Materials and Methods). The signal-to-background ratio *I*_a_/*I*_bg_, reflecting local Bcd concentration amplification within clusters, averaged 2.2 ± 0.8 across nearly 10^5^ clusters (Fig. 2B).

Given the exponential variation in nuclear Bcd concentration *I*_nuc_ along the embryo axis, we examined how cluster properties change with concentration. Cluster count strongly depended on *I*_nuc_, showing an almost twofold decrease across the anterior ∼60% of the embryo length (Fig. 2D and fig. S8), indicating less frequent clustering in nuclei with lower Bcd concentrations.

Similarly, 〈*I*_a_〉 showed a considerable dependence on *I*_nuc_, with nearly a twofold change over the same range (Fig. 2E), while 〈*d*〉 varied insignificantly (Fig. 2F and fig. S9). The mean and variance of *d* remained consistent across different *I*_nuc_ ranges (fig. S6, B and C), and there was no correlation between *d* and *I*_a_ (fig. S6A). Thus, cluster size *d* is independent of both nuclear Bcd concentration *I*_nuc_ and cluster concentration *I*_a_.

These results imply that mechanisms like droplet growth by coalescence, typical of LLPS condensates, may not apply to Bcd clusters (*33*). This speculation is also supported by the linear dependence of average cluster concentration 〈*I*_a_〉 on nuclear concentration *I*_nuc_ (*R*^2^ = 0.6) (Fig. 2E and fig. S9D), which contrasts with the switch-like dependence seen in LLPS condensates (*20*). Moreover, clustering occurs even at very low Bcd concentrations, suggesting no clear threshold for cluster formation (*34*). Further studies are needed to determine whether these clusters represent a matured state where traditional LLPS rules do not apply, or if detailed imaging of cluster dynamics is necessary to clarify these distinctions.

### Positional information of Bcd clusters

Previous work established that the position of anterior nuclei in the early *Drosophila* embryo can be determined with spatial precision better than 1% using nuclear Bcd concentration alone (*35*). This precision arises from the collective contribution of all nuclear Bcd molecules, reproducible to within 10%. Given that clusters constitute only a small fraction of nuclear Bcd molecules (fig. S10B), we investigated whether these clusters could still provide an accurate estimate of nuclear concentration and, consequently, nuclei positioning along the embryo’s AP axis.

To assess this, we considered the cluster intensity, *I*_c_, representing the molecular count of Bcd within a cluster, where *I*_c_ = 2*I*_a_σ_1_σ_2_ with σ_1_ and σ_2_ being cluster-specific parameters (Fig. 2A and Materials and Methods). Using this quantity and previous estimates for molecular count conversion (*21*), we computed the absolute number of Bcd molecules per cluster (fig. S10C).

**Fig. 3.**
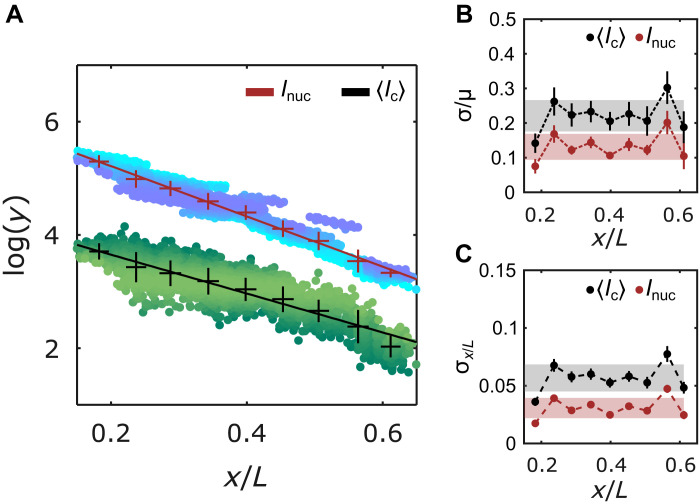
Precision of cluster positional information potential. (**A**) Overall Bcd-GFP nuclear intensity *I*_nuc_ and the nuclear average of Bcd-GFP cluster intensity 〈*I*_c_〉 as a function of nuclear position *x*/*L* (with embryo length *L*). 〈*I*_c_〉 measures the molecular count within the clusters (fig. S10 and Materials and Methods). The *y* axis is in natural logarithm units. Blue (*I*_nuc_) and green (*I*_c_) shaded data points represent individual nuclei (2027 nuclei in 14 embryos). Data are partitioned in *x*/*L* bins (mean and SD shown, error bars calculated from bootstrapping; exponential decay constants extracted from linear fits (solid lines) with $\lambda_{I_{\text{nuc}}}=0.23\pm 0.03L$ and $\lambda_{I_{c}}=0.26\pm 0.02L$). (**B**) Coefficients of variation (c.v.) σ/μ for *I*_nuc_ and 〈*I*_c_〉 as a function of *x*/*L* bins. (**C**) Errors in determination of nuclear positions using *I*_nuc_ (red) and 〈*I*_c_〉 (gray) as a function of *x*/*L* bins (obtained via error propagation, Materials and Methods). For (B) and (C), gray and red shades indicate the overall mean ± SD across all positions for 〈*I*_c_〉 and *I*_nuc_, respectively.

The nuclear average of 〈*I*_c_〉 decays exponentially with nuclear position, mirroring the *I*_nuc_ gradient: the exponential decay constants for 〈*I*_c_〉 and *I*_nuc_ are statistically similar (Fig. 3A). Thus, the molecular count of an average cluster reflects the Bcd nuclear concentration gradient. The coefficients of variation for *I*_nuc_ and 〈*I*_c_〉 are 14 ± 4% and 22 ± 4%, respectively (Fig. 3B). Despite clusters representing only a small fraction of nuclear Bcd molecules (5 to 10%, fig. S10), the 〈*I*_c_〉-derived Bcd gradient shows remarkably low variability, suggesting tightly controlled mechanisms regulating cluster formation.

As a morphogen, Bcd’s nuclear concentration provides positional identity to a nucleus with sufficient accuracy to distinguish neighboring nuclei in the anterior 60% of the embryo based on Bcd concentration alone (*35*, *36*). To estimate the positional information contained in the cluster-derived Bcd gradient, we calculated the error in position determination σ(*x*) from concentration fluctuations δc(*x*) in the gradient (*35*). Using error propagation, the positional error is given by $\sigma \left(x\right)=\delta c\left(x\right){|\frac{\mathrm{dc}\left(x\right)}{\mathrm{dx}}|}^{-1}$, where *c*(*x*) is the Bcd concentration at position *x*.

Using 〈*I*_c_〉, the positional error is σ(*x*) = 5.5 ± 0.7% of the embryo length, *L* (Fig. 3C), corresponding to a positional precision of roughly three cell diameters, which is less precise than the single-cell precision previously reported using the full nuclear Bcd concentration *I*_nuc_. Similarly, 〈*I*_c_〉 can be used to estimate the average nuclear concentration, *I*_nuc_, and near the embryo’s anterior, the error is ∼15% (fig. S9C), comparable to the variability of $I_{\text{nuc}}$ itself (Fig. 3B). This makes the average nuclear cluster concentration a reliable proxy for the overall nuclear Bcd concentration. Errors in nuclear concentration and position determination were also computed for other cluster properties, such as 〈*I*_a_〉 (representing the Bcd concentration in an average cluster) and 〈*d*〉 (representing the cluster size), but these showed higher errors than those using 〈*I*_c_〉 (figs. S9 and S11).

The positional error estimation derived from 〈*I*_c_〉 fluctuations reflects an average cluster’s properties. Individual clusters might provide positional information with varying accuracy, potentially approaching the positional accuracy given by *I*_nuc_. However, for genes to use this information, individual clusters must be associated with specific gene loci, as determined by their physical proximity, which we examine next.

### Cluster association with target genes

**Fig. 4.**
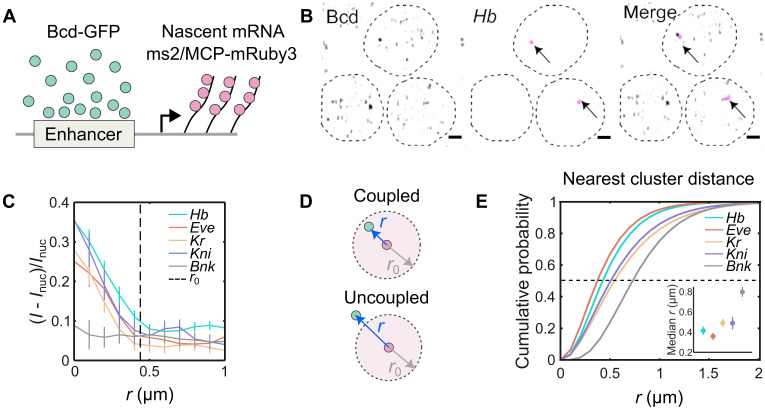
Bcd cluster colocalizes with target genes. (**A**) Cartoon showing scheme for dual color imaging with Bcd-GFP (green) and nascent transcription site labeled via the MS2/MCP system (magenta). (**B**) Images from embryos in NC14 showing nuclei expressing Bcd-GFP and *hb*-MS2/MCP-mRuby on sites of active transcription (arrows); scale bars, 1 μm. Dashed lines are a guide to the eye for nuclear boundaries. (**C**) Radial distribution of Bcd-GFP intensity around the centroid of the fluorescently labeled gene locus (i.e., hotspot). Data shown for canonical Bcd target genes, *hb* (102 nuclei, 13 embryos), *eve* (66 nuclei, 8 embryos), *Kr* (107 nuclei, 11 embryos), *kni* (90 nuclei, 6 embryos), and the nontarget gene *bnk* (56 nuclei, 10 embryos). Dashed line (*r*_0_ = 0.44 ± 0.05 μm) is twice the FWHM averaged over all genes. Data are obtained from simultaneous imaging of Bcd-GFP and MCP-mRuby3, marking the nascent transcription hotspots of the respective genes (Materials and Methods). (**D**) Schematic showing the mRNA hotspot (red) and its nearest Bcd cluster (green). When the distance *r* between the nearest cluster and the hotspot is less than the Bcd accumulation radius *r*_0_, the cluster is defined as being coupled to the gene; when it is greater than *r*_0_, the cluster is assumed to be uncoupled (see also fig. S13). (**E**) Cumulative probability distributions of distances *r* between the mRNA hotspot and its nearest cluster, computed for the same data as in (C). Dashed line is the median at EC_50_ (median effective concentration). Inset: Median distances for all genes. Errors are calculated from bootstrapping.

To understand the behavior of individual Bcd clusters near target gene transcription sites, we performed 3D imaging of labeled nascent mRNAs for the putative Bcd target genes *hb*, *even-skipped* (*eve*), *Krüppel* (*Kr*), and *knirps* (*kni*) (*37*, *38*) while simultaneously imaging Bcd-GFP (Fig. 4, A and B, and fig. S12). Bcd accumulation was observed around each target gene, with peaks of Bcd-GFP intensity at the center of the nascent mRNA hotspot (Fig. 4C). The radii of Bcd-GFP accumulation around these target genes were 490 ± 40 nm, 550 ± 20 nm, 390 ± 40 nm, and 330 ± 30 nm for *hb*, *eve*, *Kr*, and *kni*, respectively (fig. S14A). These radii were comparable to the average enrichment area radius shown in Fig. 1D (see fig. S13A for a simulation-based representation).

In contrast, no Bcd accumulation was detected around a nontarget gene, *bottleneck* (*bnk*) (*39*) (Fig. 4C), nor around nuclear centers, which serve as random sites unrelated to any specific gene locus (fig. S13B). Specificity was further confirmed by imaging NLS-GFP instead of Bcd-GFP, which showed no accumulation near the *hb* locus (fig. S13B).

These findings suggest that Bcd clusters tend to colocalize near gene loci associated with their target genes. However, Bcd clusters may not always remain directly associated with gene loci throughout the entire period of active transcription. When a Bcd cluster is not directly associated, it will be physically further from the gene transcription site (Fig. 4D). The TF accumulation radius (fig. S14, A and C) provides a distinct distance cutoff to identify clusters that are coupled to an actively transcribing gene (Fig. 4D). Thus, all the nearest clusters, whose distance from the transcription site is less than the gene’s Bcd accumulation radius, can be considered coupled to the gene.

The median 3D distances of the nearest Bcd clusters from the centers of the mRNA hotspots were determined as 420, 360, 500, and 490 nm for *hb*, *eve*, *Kr*, and *kni*, respectively, while for the nontarget gene *bnk*, it was 800 nm (Fig. 4E). Using these distance limits (Fig. 4D and fig. S14A), we applied cumulative probability plots of the nearest cluster distance distributions to calculate the fraction of transcribing genes that are coupled to a Bcd cluster (an alternate method providing similar results is shown in fig. S13C). The coupling fractions were 0.57, 0.73, 0.41, and 0.30 for *hb*, *eve*, *Kr*, and *kni*, respectively (fig. S14B). Because of its undefined accumulation radius, no such coupling fraction could be determined for *bnk*.

These results indicate that Bcd clusters preferentially localize near target genes. While both the nearest cluster distance and the fraction of coupled clusters suggest a stronger association for genes that are stronger Bcd targets, the complex architecture of these gene loci makes it challenging to directly link clustering behavior with enhancer architecture based on these findings alone. To investigate this further, we analyzed clustering using two simplified synthetic enhancer constructs that drive the expression of a reporter gene.

### Enhancer dependence of clusters

We hypothesized that if Bcd clustering depends on enhancer architecture, such as the number and affinity of Bcd binding sites, then changes in these binding site affinities should influence cluster properties. To test this, we synthesized two enhancer constructs: one with 11 high-affinity Bcd binding sites (termed the “strong enhancer”) and another with 11 low-affinity Bcd binding sites (termed the “weak enhancer”). Both constructs were designed to control a reporter gene, where an *hb* promoter drives the expression of lacZ fused with MS2 stem loops (fig. S14 and Materials and Methods).

**Fig. 5.**
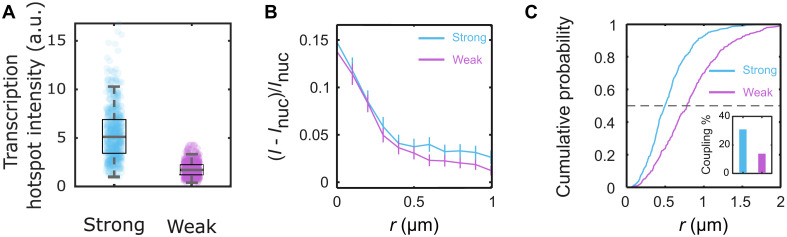
Bcd cluster colocalization is enhancer dependent. (**A**) Distributions of transcription hotspot intensities from a synthetic strong (blue, 541 nuclei, 17 embryos) and weak (magenta, 406 nuclei, 20 embryos) enhancer constructs driving an MS2-fusion reporter (Materials and Methods and fig. S14). The strong construct generates a 3.2-fold higher intensity than the weak construct, on average. Boxes represent the first and the third quartiles, while the whiskers represent the 5th and the 95th percentiles. The medians (black lines inside the boxes) are 5.1 and 1.7 for the strong and the weak enhancers, respectively. (**B**) Radial distributions of relative Bcd-GFP intensities around the centroid of the transcription hotspot. The accumulation radii are statistically identical (0.36 ± 0.05 μm and 0.39 ± 0.06 μm for strong and weak enhancer constructs, respectively). (**C**) Cumulative probability distributions of distances *r* between the transcription hotspot and its nearest Bcd cluster. The black dashed line is at EC_50_. The median distances are 0.49 ± 0.03 μm and 0.78 ± 0.05 μm for the strong and weak constructs, respectively. Inset shows the fraction of transcription hotspot coupled to a cluster for each construct (31 and 13%, respectively).

As expected, the strong enhancer resulted in a higher transcriptional output compared to the weak enhancer (Fig. 5A). However, the radial average of Bcd-GFP intensity around the transcription sites was similar for both enhancers (Fig. 5B). This finding suggests that a comparable local accumulation of Bcd is capable of producing a higher transcriptional output when driven by the strong enhancer.

The critical insight emerges when examining the properties of individual clusters. The distance from the nearest Bcd cluster to the transcriptional hotspot of the reporter gene is shorter for the strong enhancer than for the weak enhancer (Fig. 5C). This indicates that the association of individual clusters with target genes depends on enhancer strength. Supporting this, the frequency of cluster seeding is higher with the strong enhancer; approximately 31% of active transcription sites are coupled to a Bcd cluster in the case of the strong enhancer, compared to only 13% for the weak enhancer (Fig. 5C, inset).

Moreover, the intensity *I*_a_ of the nearest Bcd cluster shows a stronger correlation with the nuclear Bcd concentration *I*_nuc_ for strong enhancers than for weak ones (fig. S15). This suggests that strong enhancers are more effective at extracting positional information from the nuclear Bcd concentration gradient, potentially facilitated by the clustering process.

Given that Bcd clusters carry information about nuclear positioning, genes could access this information through interactions with the clusters. However, genes can also interact directly with the diffusing nuclear Bcd molecules to obtain the same information. Why, then, might clustering provide an advantage?

### Clusters as fast information sensors

For Bcd target gene loci to accurately interpret nuclear Bcd concentration and extract positional information from the morphogen gradient, the ability to sense diffusing TF molecules is crucial. The effective size of the sensor—whether it corresponds to a binding site (∼3 nm), an enhancer (∼50 nm), or the entire gene locus—remains unclear. Previous estimations suggest that if the binding site size is the relevant metric, then readout precision requires spatial averaging across multiple independent sensors, such as neighboring nuclei (*35*).

**Fig. 6.**
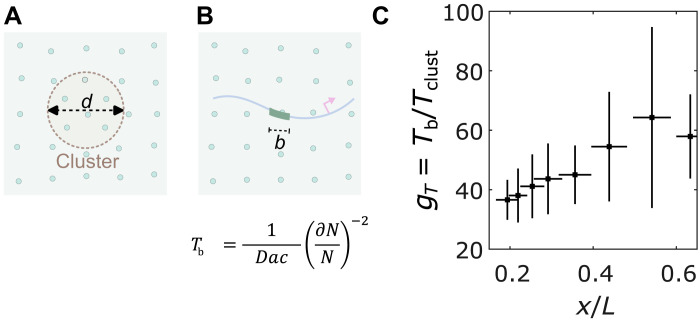
Clustering reduces time to precise concentration interpretation. (**A** and **B**) Two cartoons show Bcd molecules in the nucleus (green circles) and a cluster of diameter *d* (A) embedded in the nuclear environment and an enhancer with a binding site of length *b* (B). The equation in (B) is for the time taken by a sensor of size *a* for nuclear concentration *c* with an accuracy of $\frac{\mathrm{dN}}{N}$, where *N* is the number of molecules counted. (**C**) Reduction of time *g*_*T*_ to make an accurate (i.e., ∼10%) nuclear concentration estimation as a function of the nuclear position with the cluster as nuclear concentration sensor versus an enhancer binding site being the concentration sensor (*35*).

We propose that Bcd clusters may function as sensors, where the transcriptional output of a gene reflects the Bcd content within a cluster rather than individual interactions at target gene enhancers. Using a molecular sensing model (*35*, *40*), we treat a cluster as a sphere with diameter *d*, Bcd concentration *c*_clust_, and diffusion constant *D* (Fig. 6A). We then compare the time *T*_clust_ required for a cluster to mirror global nuclear concentration with the time *T*_b_ needed for a single binding site of size *b* to measure nuclear concentration (Fig. 6B).

The ratio *T*_b_/*T*_clust_ provides insight into their relative sensing times. If clusters act as concentration sensors, an average cluster in the anterior nucleus can sense nuclear concentration approximately 37.5 ± 5.1 times faster than a single binding site (Fig. 6C), because of its larger size and roughly twofold concentration amplification (Fig. 2B). This allows the cluster to interpret nuclear concentration in about 3 min—the timescale relevant for activating target genes (*35*, *41*). Understanding these interactions between Bcd clusters and target genes could open previously unexplored avenues for research into gene regulation and transcriptional control.

## DISCUSSION

Here, we used quantitative imaging techniques in live embryos to elucidate the role of subnuclear compartmentalization, particularly clustering, in preserving the information carried by signaling molecules within the cell nucleus. Previous research has shown that TF clusters in various organisms and tissue cultures are spatially associated with transcriptionally active sites of target genes (*13*, *15*). In contrast, according to the LLPS model, clustering results in a nonstoichiometric assembly of molecules when the global concentration exceeds a specific threshold (*20*), meaning that clusters can form in ways that do not correspond to fixed molecular ratios. This raises questions about how such clusters can maintain accurate information about nuclear concentration. More complex models, such as those involving the seeding of droplets on enhancers, also suggest that clusters do not maintain precise nuclear concentration information (*19*). Despite these challenges, it is well established that the expression of response genes is highly sensitive to the global nuclear concentrations of TFs (*35*).

To reconcile this dichotomy, we chose to study Bcd due to its unique concentration gradient, which offers two key advantages for analysis. First, its graded concentration can be optically measured with single-cell precision, providing a direct method to observe changes in nuclear concentration. Second, the transcriptional responses of multiple target genes can be dynamically measured in living embryos, allowing for real-time assessment of cluster behavior. Our experiments leverage these features and demonstrate that the intensity and total number of Bcd molecules in clusters effectively preserve nuclear concentration information.

These results prompt two fundamental questions about TF clusters. First, how do these clusters form in a way that retains information about concentration? Second, how are the features of these clusters, which convey concentration information, interpreted? Several observations from our study point to interesting areas for future research in these directions.

Any analysis of cluster formation must take into account that each cluster defines a transcriptional microenvironment integrating multiple interacting components, such as mediator molecules, chromatin-modifying agents, and Pol II (*42*–*45*). Bcd has been shown to interact with several of these components via its activation domain (*46*). Any one of these components may play a central role during the initial stages of cluster formation. Therefore, the formation and effective size of such clusters might reflect the presence of other, or indeed, all constituent molecules rather than being dependent solely on the concentration of a single molecular species like Bcd. This could explain why the average cluster size remains unchanged despite variations in Bcd nuclear concentration.

Bcd interacts with DNA through its DNA binding motif (*47*), of which multiple copies are present within each enhancer, serving as TF binding sites. Bcd’s binding to enhancers might seed cluster formation in ways that do not maintain a direct dependence on its concentration, although that concentration determines the intensity of Bcd’s accumulation in clusters. Our observation that clusters of finite sizes were present even at very low Bcd concentrations is consistent with previous studies reporting clustering at low concentrations (*28*, *48*). This contradicts what might be expected of classic single-molecule LLPS assemblies (*19*, *20*, *28*), where frequency and size are expected to depend more directly on absolute concentration. Overall, our results suggest that multiple molecular species drive cluster formation and highlight the potential role of enhancers in cluster seeding.

Techniques such as fluorescence correlation spectroscopy (FCS) (*25*, *26*) and single-particle tracking (SPT) (*29*) have been used to estimate the fraction of Bcd molecules undergoing slow diffusion. However, this fraction varies depending on the definition of slow diffusion used in each study. In our study, we detected clusters with an average size of approximately 0.4 μm that remain stable for at least 1 s, capturing only a subset of the molecules identified as slowly diffusing (*26*). While this does not rule out the existence of smaller, highly transient clusters, we likely observe only the “slowest” fraction of moving particles (fig. S10B). In contrast, the rest of the slow fraction might result from transient interactions with clusters or nonspecific binding. Fluorescence recovery after photobleaching and FCS studies (*27*) have found that only about 5% of Bcd molecules constitute the immobile fraction.

A fundamental question arising from our observations is how the Bcd cluster concentration establishes and maintains a linear relationship to nuclear concentration. Addressing this requires examining the rate at which molecules approach the cluster boundary and how intranuclear diffusion parameters influence this rate. For stable clusters, the capture rate of molecules within the boundaries must balance the escape rate. The approach rate depends on the concentration-dependent diffusion properties of the molecules. However, the relationship between molecular escape rate from clusters and concentration remains unclear. Further exploration is needed to understand how concentration information is transmitted to the clusters.

Approximately 90 to 95% of the Bcd protein in the nucleus is not in detectable clusters. Previous analysis (*35*) suggests that interpreting position based on this soluble fraction would be too slow to account for the observed dynamics and precision of transcription. We propose that the increased concentration and larger size of clusters facilitate the response of target genes to the Bcd gradient, potentially offering a mechanism to read out concentration information more quickly and accurately.

It remains unclear whether enhancers read concentration information directly from the clusters or merely serve as a medium for seeding clusters. If enhancers only seed clusters, the information content of the clusters could be interpreted directly or indirectly by the gene’s promoter region. This implies that information transmission from the cluster to the promoter depends on their physical proximity (*49*, *50*), making it an event limited by chromatin dynamics. Recent studies tracking clusters associated with transcriptional hubs have shown a correlation between cluster-promoter interaction and transcriptional burst enhancement (*44*).

Whether cluster concentration affects the frequency or duration of interactions with gene promoters remains an open question that requires careful quantitative studies. Preliminary evidence suggests no simple, direct relationship between cluster concentration and the frequency or duration of these interactions (*14*). In addition, the transcriptional microenvironment is highly complex, with multiple enhancers potentially interacting and communicating with the gene promoter via a single cluster (*10*). Therefore, understanding how information stored in clusters is relayed to specific DNA elements within this intricate microenvironment remains a serious challenge.

Using a simple model (*40*) and our measured cluster properties, we demonstrated that clusters could potentially function as concentration sensors at much faster timescales. This finding underscores a potentially crucial role for clustering in defining biological timescales, particularly for transcriptional regulation. Specifically, clustering may offer an alternative explanation for noise suppression in molecular concentration readout, traditionally attributed to spatial and temporal averaging over considerably longer timescales (*35*, *51*). Our results suggest that clusters might provide a faster mechanism for achieving this precision.

Our results indicate that Bcd clusters could be a high-fidelity mechanism for encoding and transmitting concentration information. However, the relationship between cluster formation and nuclear concentration is complex, as clusters represent only a small fraction of the total Bcd molecules in the nucleus, and their size remains invariant with concentration. Fully understanding this relationship will require examining how molecules dynamically interact within clusters and how clusters maintain a stable composition over time.

While our study is based on the behavior of a specific TF in a model organism, the principles uncovered here—such as the role of clustering in maintaining concentration information and enhancing transcriptional responses—are likely relevant to other biological systems, including mammals. For example, similar clustering mechanisms may be fundamental in developmental gene regulation, cellular differentiation, and stress responses across diverse species.

In conclusion, this study provides quantitative insights into the properties of TF clusters and their role in transmitting information from nuclear concentrations to gene loci. Moving forward, it will be critical to explore the molecular mechanisms governing cluster formation and maintenance, the dynamics of molecular interactions within clusters, and the exact pathways through which clusters influence transcription. Advanced imaging techniques, such as single-molecule tracking and high-resolution fluorescence microscopy, complemented by sophisticated computational models, will be essential for this research. Such efforts promise to expand our understanding of gene regulation across a wide array of biological contexts, potentially leading to innovative therapeutic strategies for diseases where gene expression is dysregulated.

## MATERIALS AND METHODS

### Fly husbandry and genetics

*Drosophila* fly lines expressing bcd-GFP from (*36*) were used as the starting point. In all such lines, the endogenous b*cd* was replaced with a null phenotype *bcd^E1^*. Stable stocks expressing NLS-MCP-mRuby3; b*cd*-eGFP-*bcd^E1^* were created. Virgins from these stocks were then crossed with males expressing reporter constructs with the gene regulatory regions, while the gene body was substituted with MS2 stem-loop cassettes and LacZ.

For the synthetic enhancers, the following scheme was used: A 472–base pair fragment spanning the modified *hb* proximal enhancer and the *hb* P2 basal promoter was synthesized by IDT and ligated into the piB-hbP2-P2P-MS2-24x-lacZ-αTub3′UTR construct (*52*) between the restriction sites HindIII and NcoI. In the resulting reporter construct, the hb promoter drives the expression of 24 copies of the MS2 loops and is followed by the lacZ coding sequence. The number of MS2 loops in the reporter was verified by Sanger sequencing. In the strong enhancer reporter, eight suboptimal Bcd binding sites were converted to the consensus sequence TAATCC, resulting in a total of 11 strong Bcd binding sites. In the weak enhancer reporter, all three consensus sequence TAATCC were converted to the suboptimal Bcd binding site TAAGCT, resulting in a total of 11 weak Bcd binding sites. Both constructs were integrated into the 38F1 landing site on chromosome II of the fly line FC31 (y+); 38F1 (w+) using FC31 integrase-mediated cassette exchange (*53*). All fly lines from which males were crossed and their sources are tabulated in table S1.

### Sample preparation

Embryos were harvested on apple juice plates, using protocols mentioned earlier (*36*). Staged 2-hour-old embryos were dechorionated by hand by rolling them over a tape band (Scotch). Dechorionated embryos were placed on the lateral side on a mounting membrane lined with glue. The glue was prepared by submerging 10 cm of Scotch tape in 4 ml of heptane for 48 hours in a shaker at 37°C. A drop of glue was placed on the mounting membrane, gently smeared evenly, and was then allowed to air dry before placing the dechorionated embryos. After the embryos were placed on the membrane, they were submerged in a mixture of halocarbon oil (60% Halocarbon 27 and 40% Halocarbon 700, Sigma), and then covered with a 25 × 25 mm^2^ glass coverslip (Corning).

### Imaging

3D stacks of fluorescence images were acquired using the fast Airyscan mode of a Zeiss LSM 880 microscope, run by Zen Black 2.3, SP1 software. A Plan-Apochromat 63×/1.4 oil immersion objective (Zeiss) was used for all measurements. GFP was excited with the 488-nm line of the Argon laser (140 μW), while mRuby3 was excited using the 561-nm diode-pumped solid-state laser (36 μW). Laser power at the back aperture of the objective was measured with a power meter (PM100D, Thorlabs) at the beginning of each measurement session. The MBS 488/561 beamsplitter combined the beams. The emission filter set, BP 420 to 480/BP 495 to 550 was used for GFP emission, while BP 495 to 550/LP 570 was used for mRuby3. The effective emission peak wavelengths were 515 nm for GFP and 578 nm for mRuby3. A detector gain of 740 was used for all imaging cases. The voxel size was fixed at 43 × 43 × 200 nm^3^ for all 3D measurements. For 2D single-plane videos, however, the z-section thickness was 1000 nm. The frame times were 497 ms for each frame for both color channels, with a pixel dwell time of 0.744 μs. Each image frame was 1044 × 1044 pixels, or 45 × 45 μm for the 3D acquisitions. No averaging was done. Imaging was done using the “Fast Airyscan” mode, with final images obtained after applying the “Airyscan Processing” within the Zen software.

Imaging was conducted on embryos in nuclear cycle no. 14, between the 20th and the 35th minute after mitosis. The nuclei at the embryo’s surface facing the glass coverslip were imaged. To ensure that the entire nucleus was scanned, a total z depth of 14 μm, with the central plane of the nucleus as the center was imaged. The stack was split into 70 z-frames, with a ∼500-nm frame thickness. The horizontal dimensions of the images were ∼45 × 45 μm along the *x*-*y* plane, spanning ∼40 nuclei. Four such image stacks were recorded per embryo at various positions along the anterior-posterior (AP) axis.

### Embryo fixation

Embryo fixation presented two challenges: (i) preserving the fluorescence of GFP after fixation, and (ii) preserving the clusters themselves. To address both, we exclusively used freshly dissolved methanol-free formaldehyde (Thermo Scientific Pierce) at a final concentration of 4% for embryo fixation. Throughout fixation and handling, we ensured that the embryos’ exposure to organic solvents such as heptane, methanol, or ethanol was minimal. With these modifications to the standard protocol (*54*), fixation and visualization of Bcd clusters in the embryos can be achieved.

### Pixel correlation

To achieve pixel correlation, we separately autocorrelated the pixels along the *x* and *y* axes. We used the crosscorr function in MATLAB for this purpose. Although this function is typically used to determine the similarity between a time series and a lagged version of another series, in our case, we adapted it to find the autocorrelation of a pixel row (or column) with a lagged version of itself. For pixel rows, (*x*), we get the correlation function (*c*) to be

$$c_{x,x}=\left\{ \begin{array}{cc} \frac{1}{T}\sum_{t=1}^{T-k}\left(x_{n}-\bar{x}\right)\left(x_{n+k}-\bar{x}\right) & k=0,1,2,\ldots \\ \frac{1}{T}\sum_{t=1}^{T-k}\left(x_{n}-\bar{x}\right)\left(x_{n-k}-\bar{x}\right) & k=0,-1,-2,\ldots \end{array}\right.$$ (1)

This is repeated over all the rows and the average is then calculated. The pixel columns (*y*) were similarly treated, after which the averages of the rows and columns were calculated. The correlation lengths calculated along the *x* axis were equal to those calculated along the *y* axis for all images. The *x*- and *y*-axis data were then combined to obtain the overall image average. The average function was fitted with an exponential, y(x)=a+b⋅exp(−c⋅x), and the “correlation length” was computed by $\lambda_{\text{corr}}=x_{0}+\frac{\text{log}\left(2\right)}{c}$. Subsequently, the error in the correlation length is given by $\sigma_{\lambda }=\lambda \cdot \frac{\sigma_{c}}{c}$. This operation is selectively done for either the pixels exclusively within or outside the nuclear masks in the images.

### Local maxima detection

High-intensity foci of GFP-tagged proteins are scattered throughout the nucleus. Some of these foci result from protein clustering, while others are due to noise in the intensity. The centroids of these foci appear as local intensity maxima, and detecting them involves a two-step process. While the first step is applied only once, the second step is iteratively applied until the local maxima are located with high accuracy.

In the first step, the nuclear pixels are segmented and an Otsu thresholding is performed. Only pixels with values above the threshold are retained, while the rest are converted to “not a number” (NaN). The nuclear pixels are then rescaled to the interval [0, 1], resulting in image *I*^1^ (fig. S4A, top), to which the second step is applied.

In the second step, local thresholding is applied. First, a 25 × 25 pixel window is created and the moving mean (μ_*k*_) and moving SD (σ_*k*_) are computed using the window on the nuclear pixels. This results in two matrices, one containing the moving means, μ_*I*^1^_ (fig. S4A, middle row, left) and the other containing the moving SDs, σ_*I*^1^_ (fig. S4A, middle row, right), which are added (μ_*I*^1^_ + σ_*I*^1^_). This sum serves as the local threshold matrix, which is subtracted from *I*^1^. Pixels in *I*^1^ with values below the corresponding cell in the local threshold matrix ($\mu_{I^{1}}^{}+\sigma_{I^{1}}^{}$) are set to zero and the resulting image is rescaled to the interval [0, 1] (fig. S4A, bottom row). This gives the image, $I_{2}$, from which moving mean and moving SD matrices are calculated and a new threshold matrix is generated ($\mu_{I^{2}}+\sigma_{I^{2}}$). This threshold matrix is subtracted from $I_{2}$ to obtain $I_{3}$ (fig. S4B, bottom row). These steps are iteratively applied *m* times yielding a set of images *I*^1^…I^*m*^. With each local thresholding iteration, fewer pixels are retained around the local maximum, determining the center of the local maximum more accurately with each iteration.

To determine the optimal *m* iterations required for optimal maxima localization, we first binarized the images $I^{i\in \left[1,m\right]}$ by setting all nonzero pixels to 1. In the resulting binarized images, $I_{\text{bin}}^{i\in \left[1,m\right]}$, we calculated the structural similarity index (SSIM) values using the built-in MATLAB function ssim, to assess the differences introduced in the images as a result of local thresholding. Specifically, we computed pairwise SSIM values of the images $I_{\text{bin}}^{i}$ with respect to the image, $I_{\text{bin}}^{1}$ as the reference image. The SSIM value drops with each i∈[1,m], as the subsequent images are progressively poorly correlated with the starting image. However, at m∼15, the SSIM values stabilized, indicating that further local thresholding would not improve the maxima detection.

In the subsequent image, $I_{\text{bin}}^{m}$ was used to compute the location of the centroids of the local maxima. This gave us the location of the intensity maxima in the nuclei with very high precision, although the maxima detected cannot be sorted by the size of the corresponding spots.

### Pair correlation

The local maxima in the nuclei are identified in all the frames of a video, and subsequently projected into a single map. The resulting time projection of the Bcd-GFP local intensity maxima has randomly dispersed points and focal accumulations of points in space. The randomly distributed points can be considered representative of a Poisson process and the focal accumulations can be modeled as Gaussian functions convolved with hypothetical singularities. To estimate the average density and effective size and the relative density of maxima within these focal accumulations representing a Gaussian process, we use the pair-correlation function (*32*).

The density function of the points expressed in polar coordinates is given by $\rho \left(\vec{r}\right)$. The pair-correlation function for such a point distribution is given by Veatch *et al.* (*32*)

$$g\left(\vec{r}\right)=\langle \rho \left(\vec{R}\right)\rho (\vec{R}-\vec{r})\rangle /\rho^{2}$$ (2)

Here, ρ is the average density. In practice, this correlation function is calculated using fast Fourier transforms applied to an image *I* containing the point distribution

$$g\left(\vec{r}\right)=\frac{1}{\rho^{2}}\cdot \frac{{\text{FFT}}^{-1}\left[\text{FFT}{\left(I\right)}^{2}\right]}{{\text{FFT}}^{-1}\left[\text{FFT}{\left(W\right)}^{2}\right]}$$ (3)

Here, *I* is a sparse matrix with 1 s at the locations of the maxima and 0 s elsewhere. The quantity *W* is a window matrix adjusted to fit within the area of a nuclear cross section taken as a convex hull.

If we consider the density of the Poisson process to be 1, and the Gaussian process peak density to be ρ′ above 1, we get the expression

$$g\left(r\right)=\rho \prime \text{exp}\left(-r/\sigma^{2}\right)+1$$ (4)

Here, σ denotes the size of the focal accumulation of the maxima, representing the Gaussian processes. This expression can then be used to fit the pair-correlation function to derive the effective width of the function and infer the increase in density within these Gaussian accumulations.

### Positioning a nucleus in the embryo

To determine the position of a nucleus in the embryo, we define a coordinate system. Initially, two images are acquired to get the full 2D extent of the embryo: one of the embryo’s anterior and one of the posterior halves, imaged at the midsagittal plane with otherwise identical imaging conditions as for the nuclei. From these two images, we construct the compound image of the full embryo and identify (in software) the locations of the anterior $\left(x_{0},y_{0}\right)$ and posterior $\left(x_{L},y_{L}\right)$ tips of the embryo (*L* is the length of the embryo) in microscope stage coordinates.

The line connecting these two points represents the A-P axis, or the *x* axis in the embryo coordinate system (X′). Perpendicular to this line is the *y* axis (Y′), passing through $\left(x_{0},y_{0}\right)$. Hence, the anterior end is (0,0) and the posterior end is (L,0) in the embryo coordinates.

Now, if the centroid of a nucleus is ($x_{i},y_{i}$) in the microscope stage coordinates, the distance of the nucleus from $\left(x_{0},y_{0}\right)$ is $r_{i}=\sqrt{{\left(x_{i}-x_{0}\right)}^{2}+{\left(y_{i}-y_{0}\right)}^{2}}$ and the angle made by $r_{i}$ with the A-P axis is given by $\theta_{i}=\text{arctan}\left[\left(y_{i}-y_{0}\right)/\left(x_{i}-x_{0}\right)\right]-\text{arctan}\left[\left(y_{L}-y_{0}\right)/\left(x_{L}-x_{0}\right)\right]$. Hence, the location of a nucleus in the X′ coordinates is given by $x_{i\prime }=r_{i}\cdot \text{cos}\left(\theta_{i}\right)$, or

$$x\prime_{i}=\sqrt{{\left(x_{i}-x_{0}\right)}^{2}+{\left(y_{i}-y_{0}\right)}^{2}}\cdot \text{cos}\left[{\text{tan}}^{-1}\left(\frac{y_{i}-y_{0}}{x_{i}-x_{0}}\right)-{\text{tan}}^{-1}\left(\frac{y_{L}-y_{0}}{x_{L}-x_{0}}\right)\right]$$ (5)

This value can be computed for each nuclear centroid. To pool nuclei by their position, nuclei with $x_{i\prime }$ within the position bin edges are accumulated.

### Segmentation of nuclei using Bcd-GFP intensity

Nuclei segmentation is based on the GFP signal in Bcd-GFP–expressing embryos, which reduces the number of segmentable nuclei to those that are GFP enriched, or “filled,” such that the nuclear boundary can be accurately identified using the higher intensity of GFP within the nucleus. Automated segmentation of Bcd-GFP–expressing nuclei was done using the following scheme: First, the raw images were contrast adjusted using imadjustn, then filtered with a median filter, medfilt3, followed by a Gaussian filter, imgaussfilt3. A cuboidal structural element was then used for a series of morphological transformations to the resulting images. Erosion was applied (imerode), followed by a reconstruction (imreconstruct), and then dilation (imdilate). The complement of the reconstructed image was obtained (imcomplement) and then blurred with a Gaussian filter (imgaussfilt3). The resulting image was closed (imclose) and eroded, and a binary mask for the nucleus pixels was subsequently obtained. Last, watershed segmentation (watershed) was applied to separate any conjoined neighboring nuclei. Labels were then assigned to the nuclear masks to identify individual nuclei.

### Locating clusters (in 3D)

A technique for 2D local maxima localization was introduced in the “Local maxima detection” section. This technique indiscriminately detects all local intensity peaks, including noise spikes and protein clusters. The difference between a “real” cluster and a “noise-related local maximum” is that the spot size for a cluster is at least as large as the PSF, while a noise-related maximum is likely to be smaller. To capture this difference, we chose a pixel size smaller than the Nyquist criterion of a diffraction-limited spot. With an *x*-*y* size of 43 nm, a diffraction-limited spot spans 4 to 5 pixels in either direction. Hence, a cutoff limit of 3 pixels effectively differentiates a cluster from a noise-related maximum. For z-slices, we chose a thickness of 200 nm. Because the PSF width along the *z* direction is larger than 500 nm, any maxima that do not span at least two z-slices are likely noise-related maxima. Hence, we chose 2 pixels as the cutoff limit along the *z* axis.

For live imaging of mobile structures like subdiffusive clusters, the likelihood of detecting a cluster in two consecutive frames depends on the frame rate. As shown in fig. S5H, the detection probability of a cluster in frames imaged ∼500 nm apart is greater than 70%. Therefore, for z-slices imaged 200 ms apart with thickness less than the PSF width (∼500 nm), we should be able to detect the cluster with high reliability.

To identify only relevant puncta-like entities in the nucleus, we used the following technique on the raw images of Bcd-GFP nuclei. First, morphological top-hat filtering was applied using a “disk” as the structural element to the 3D raw images of the nuclei using top-hat filtering. The transformed image thus obtained was used to detect local intensity maxima peaks. For this, the top 1 percentile pixels within a nucleus were selected from the transformed images. Joined neighboring spots were then separated by applying a watershed algorithm.

It can be argued that the centroid of the local maxima peaks from the raw images is preserved through this morphological transformation. Next, the spot mask is obtained from the spot segmentation in the morphologically transformed image, and the mask is then applied to the raw image. Intensity-weighted centroids in 3D of the voxels within each mask are then calculated using WeightedCentroid on the raw image. This gives the peak position of each cluster.

However, not all clusters thus detected are retained for further analysis. A size thresholding (as mentioned above) is then performed such that if the *x*-*y* cross section of a detected spot is less than 3 × 3 pixels wide and the z depth is not at least 2 pixels wide, the spot is discarded. That brings the threshold volume to 3 × 3 × 2 = 18 pixels. A corresponding effective spot diameter *d* can be calculated from the threshold volume, such that $d={\left(6/\pi \times \text{vol}\right)}^{1/3}$. The threshold diameter turns out to be 3.25 pixels wide, which, converted to absolute units, gives ∼140 nm, which is substantially less than the 3D PSF of the microscope. Thus, using this technique, we identify the locations (only) of the “real” puncta in the Bcd-GFP nuclei.

### Fitting clusters

To extract cluster-relevant parameters, cluster fitting is performed on the raw image pixels. Although a cluster is a 3D entity spanning multiple imaging sections, we perform a 2D fitting of the intensity profile in the plane passing through the intensity-weighted center along the *z* axis. Given that the resolution along the *z* axis is approximately five times poorer than along the *x*-*y* axes, any fitting along the *z* axis introduces larger errors. Pixels within a square window centered on the cluster centroid are chosen from the plane of the cluster centroid’s z-coordinate. The window length is set at 2*w* + 1 pixels, where *w* is ∼12 pixels for a typical window size of 1.1. × 1.1 μm^2^.

To fit the intensity profile within this window, a 2D Gaussian function is used (*55*). The fitting procedure uses least-square curve fitting (lsqcurvefit) with the Levenberg-Marquardt algorithm. The Gaussian fitting equation used is

$$\begin{array}{c} f\left(x,y\right)=I_{a}\text{exp}\{-[a{\left(x-x_{0}\right)}^{2}+\\ 2b\left(x-x_{0}\right)\left(y-y_{0}\right)+c{\left(y-y_{0}\right)}^{2}]\}+I_{\text{bg}} \end{array}$$ (6)

where

$$\begin{array}{c} a=\frac{{\text{cos}}^{2}\theta }{2\sigma_{1}^{2}}+\frac{{\text{sin}}^{2}\theta }{2\sigma_{2}^{2}}\\ b=\frac{\text{sin}2\theta }{4\sigma_{1}^{2}}+\frac{\text{sin}2\theta }{4\sigma_{2}^{2}}\\ c=\frac{{\text{sin}}^{2}\theta }{2\sigma_{1}^{2}}+\frac{{\text{cos}}^{2}\theta }{2\sigma_{2}^{2}} \end{array}$$ (7)

Here, $I_{a}$ represents the intensity amplitude, and $I_{\text{bg}}$ is the background intensity level. The initial guess value for $I_{a}$ was the pixel value at the center of the window, and $I_{\text{bg}}$ was approximated as $I_{\text{nuc}}$, the average intensity of the nucleus. The initial guesses for both $\sigma_{1}$ and $\sigma_{2}$ are w/2, and the rotational angle θ is initialized to *0*. Bounds for $\sigma_{1}$ and $\sigma_{2}$ are set to $w^{2}$ and the bounds to $x_{0},y_{0}$ are set to −w:w. The angle θ is constrained between 0<θ<π/4. Candidates whose fitted parameters do not meet the criteria are automatically discarded as cluster candidates (<2%). It is important to note that $I_{a}$ and $I_{\text{bg}}$ are obtained separately from the fits, ensuring that $I_{a}$ is automatically background corrected.

### Cluster properties

Using the parameters obtained from the fits, we obtain the measures for three cluster properties: cluster size, *d*, the concentration of Bcd molecules within a cluster, and the total molecules inside a cluster. For the notations used in this section, refer to the parameters obtained in the previous section.

To calculate the effective size of clusters, we consider the Gaussian spread along *x* and *y* directions ($\sigma_{x},\sigma_{y}$). The effective radius, $r_{\text{eff}}$, is derived as $r_{\text{eff}}=\sqrt{\sigma_{1}^{2}+\sigma_{2}^{2}}$. This represents the effective size of the 3D cluster, assuming the Gaussian width as a projection on a section of the obloid-shaped cluster representing the actual spot.

The intensity amplitude $I_{a}$ of the cluster gives an estimate for the concentration of Bcd molecules in the cluster. To obtain an estimate for the total number of molecules within a cluster, we consider the quantity $I_{c}=2\pi I_{a}\sigma_{1}\sigma_{2}$.

After obtaining the measures for the cluster properties, compute their nuclear averages, $\langle d\rangle ,\langle I_{a}\rangle ,\langle I_{\text{bg}}\rangle$, and $\langle I_{c}\rangle$. These nuclear averages of the measures of cluster properties are then examined for correlation with the nuclear Bcd concentration, given by $I_{\text{nuc}}$, and the position of the nucleus in the embryo x/L. Subsequently, the nuclear average cluster property values are discretized into equidistant bins of either the normalized nuclear positions (x/L) or the average nuclear concentration ($I_{\text{nuc}}$). For each bin, an equal number of bootstrap data samples are drawn and the mean and the SDs are separately computed.

### Slope calculation

The nuclear averages of the measures of cluster properties, $\langle d\rangle ,\langle I_{a}\rangle ,\langle I_{\text{bg}}\rangle$ and $\langle I_{c}\rangle$ (or their natural logarithms) are plotted against the corresponding nuclear Bcd concentration, $I_{\text{nuc}}$ (or the nuclear position, x/L). Linear regression models are fitted to the data, and parameters such as the coefficient of determination $R^{2}$, the slope of the linear fit, and the error in the slope are derived from these models.

The nuclear averages of the measures of cluster properties are assumed to linearly correlate with $I_{\text{nuc}}$, whereas their dependence on x/L follows an exponential pattern. Therefore, the slope of the linear fit of the natural logarithm of the nuclear averages of the measures of cluster properties, plotted against x/L, can be used to determine the exponential decay constant λ, such that $\lambda =-\left(1/\text{slope}\right)\pm \sigma_{\text{slope}}/{\text{slope}}^{2}$.

### Error in nuclear property estimation using cluster property

To estimate the error in nuclear concentration estimation $\sigma_{c}$ using cluster properties like $\langle I_{a}\rangle$, 〈d〉, and $\langle I_{c}\rangle$, we use the slope *s* obtained from the linear fits and the error in the cluster property estimation in each concentration bin $\sigma_{i}$. The formula for $\sigma_{c}$ is derived as $\sigma_{c}=\sigma_{i}/s$. In addition, the uncertainty associated with each $\sigma_{c}$ is $\sigma_{\sigma_{c}}=\sigma_{c}\cdot \sqrt{{\left(\sigma_{\sigma_{i}}/\sigma_{i}\right)}^{2}+{\left(\sigma_{s}/s\right)}^{2}}$, where $\sigma_{\sigma_{i}}$ represents the error associated with cluster property determination, and $\sigma_{s}$ denotes the error in the slope (*s*). Similarly, the error in estimating nuclear position, $\sigma_{p}$ using cluster properties is determined using the exponential decay constant, λ as $\sigma_{p}=\lambda \cdot \sigma_{i}/i$, where λ represents the exponential decay constant and *i* denotes the average nuclear position. The uncertainty in $\sigma_{p}$ is given by $\sigma_{\sigma_{p}}=\sigma_{p}\cdot \sqrt{{\left(\sigma_{\sigma_{i}}/\sigma_{i}\right)}^{2}+{\left(\sigma_{i}/i\right)}^{2}+{\left(\sigma_{\lambda }/\lambda \right)}^{2}}$. All these expressions are derived from the laws of error propagation.

### Segmentation of nuclei using MCP-mRuby3 intensity

While Bcd-GFP enriches the nucleus, the relative intensity of MCP-mRuby3 is higher in the cytoplasm than in the nucleoplasm, making the nuclei appear “hollow.” Segmentation of MCP-mRuby3–expressing nuclei involved an approach distinct from Bcd-GFP–expressing nuclei. Because MCP-mRuby3 nuclei lack fluorophores, their signal is lower than that of the internuclear space. To segment these nuclei, we first adjusted the image brightness using imadjustn, which increases image contrast by adjusting intensity values. Next, we applied a 3D median filter followed by a Gaussian filter. An extended regional maxima transformation (imextendedmax) was then performed. The resulting image was binarized, and the inverse of the binary image was created. Subsequently, a 3D kernel was convolved with the binary image, and a threshold was applied. Watershed segmentation was applied to the thresholded image. The resultant image underwent opening using a cuboidal structural element, and any holes within the bright structures were filled to generate the final mask for the nuclei.

To match a nucleus imaged through the Bcd-GFP channel with that imaged through the MCP-mRuby3 channel, we checked if their centroids were within half a nuclear length of each other. Nuclei that could not be mapped in this manner were discarded from further analysis.

### Transcription hotspot detection

Transcription hotspots are nascent mRNA accumulations at the site of active transcription in the nucleus. These nascent mRNA molecules have MS2 stem loops that are bound by MCP-mRuby3 fusion proteins. The mRuby3 fluorescence intensity lets hotspots appear as bright spots within the nucleus against a darker background. To identify these spots, the nuclear boundaries were determined using a hollow nuclear segmentation approach (see above). Within these boundaries, the identification of transcription hotspots began with the application of a Difference of Gaussian (DoG) algorithm to the raw images. The resulting image was convolved with the original raw image and then rescaled. A threshold based on the nuclear pixel intensity ($I_{\text{nuc}}$) was applied, discarding any pixels with intensities lower than $4\sigma_{I_{\text{nuc}}}+\mu_{I_{\text{nuc}}}$. Subsequently, masks representing potential transcription hotspots were generated, and a size cutoff of 18 pixels was imposed on these masks.

### Radial intensity profile and coupling fraction calculation

To calculate the distance limits for a Bcd cluster to be coupled with an mRNA hotspot, we first calculate the width of the radial intensity profile of Bcd-GFP around the mRNA hotspot. Bcd-GFP clusters associated with a transcriptional hotspot might coincide with the hotspot, in which case the distance is given by the centroid distances of the two fluorescence accumulations.

First, the mRNA hotspots were segmented, and their intensity-weighted centroids were determined by using the regionprops function of MATLAB. Next, the intensity of Bcd-GFP ($I_{r}$) was computed, *r* being the radial distance from the transcription hotspot centroid. The intensity profile was computed exclusively on the *x*-*y* plane passing through the hotspot centroid. $I_{r}$ was obtained by averaging pixel intensities within a ring from *r* to *r* + 0.1 μm. Data for each $I_{r}$ were aggregated across multiple nuclei from various embryos to generate an average $I_{r}$ profile [fig. S13, B and C (cyan, error bars)].

To determine the accumulation radius for a gene, the average $I_{r}$ profile was fitted with a double Gaussian function [fig. S13C (cyan, solid line)]

$$f\left(x\right)=k_{0}e^{{\left[-\frac{\left(x-x_{0}\right)}{a_{0}}\right]}^{2}}+k_{1}e^{{\left[-\frac{\left(x-x_{1}\right)}{a_{1}}\right]}^{2}}$$ (8)

Here, $r_{0}$, the accumulation radius, was defined as the full width at half maximum (FWHM) of the first Gaussian component. The error in $r_{0}$ was calculated from the fitting error.

Furthermore, the distance from the intensity-weighted centroid of the nearest Bcd-GFP cluster to the transcription hotspot centroid was measured. A histogram of these distances was plotted, and the cumulative probability function was derived directly from the histogram or through spline fitting. The cumulative probability value at $x=r_{0}$ provided the fraction of active transcription sites coupled to a cluster (Fig. 5C).

Alternatively, the histogram of the nearest neighbor TF cluster distances from the mRNA hotspot could be fitted with double Gaussians [fig. S13C (purple, broken lines)]. The first peak corresponded to the nearest neighbor cluster coupled to the gene, and the second, weaker peak indicated the cluster nearest to the gene that was not coupled. The intersection of these two Gaussian fits marked the boundary ${r^{\prime }}_{0}$ [fig. S13C (black, broken lines)], ensuring only clusters inside this boundary were considered coupled.

The radius $r_{0}$ is influenced by several factors that contribute to broadening. Chromatin, not being stationary but subdiffusive in the nuclear space, causes motion blurring of point sources during video capture. In addition, mRNA hotspots, consisting of multiple MS2 stem loops spread across the gene body, can span several kilobases at any given moment. Moreover, the stem loops project out of the gene body with their own degrees of freedom. Transcription, being a kinetic process, allows stem loops to traverse linearly along the gene body at the speed of transcription (approximately 2 kilobases/min). These factors collectively broaden the MS2 hotspot signal, necessitating its approximation as a point source convolved with a Gaussian to incorporate all forms of broadening.

### Estimation of molecules per cluster and the total cluster fraction

In a previous study, the total number of Bcd molecules in the nucleus was estimated using Western blots (*21*). However, the construct was such that the Bcd concentration was uniform throughout the embryo, unlike the exponential decay observed along the axis in the wild-type gradient. Given that the total number of Bcd molecules in the flat expression lines remains equivalent to the total expressing molecules in the wild-type gradient, we can establish a relationship between these quantities as follows

$$N_{0_{\text{flat}}}\int_{0}^{L}\;\mathrm{dx}=N_{0}\int_{0}^{L}e^{-x/\lambda }\;\mathrm{dx}$$ (9)

Here, $N_{0_{\text{flat}}}$ represents the average concentration of Bcd in a nucleus in the flat expression lines used in the study, while $N_{0}$ denotes the concentration at *x* = 0 for the wild-type line, which exhibits an exponential gradient with a length constant λ.

Solving this equation yields $N_{0}=N_{0_{\text{flat}}}L/\lambda$. Using approximate values $N_{0_{\text{flat}}}∼8000$, and λ∼0.2, we obtain $N_{0}\equiv 40,000$ molecules. Assuming an average nuclear diameter of 5 μm, the average density of Bcd molecules in the nucleus is approximately 600 molecules/μm^3^.

Given that an average cluster has a concentration of Bcd that is 2.2 times higher than the nucleoplasm, the Bcd concentration inside a cluster at the anterior of the embryo is 1320 molecules/μm^3^. With an average cluster diameter of ~0.4 μm, the average volume is $0.03\;\mu m^{3}$. By knowing the molecular density within a cluster and its volume, we calculate that there are approximately 37 molecules per cluster at the anterior of the embryo. An estimate of the molecules per embryo along the embryo axis is shown in fig. S10C.

### Derivation of the concentration sensing limit

We consider a spherical cluster with an effective diameter *d*, containing a concentration of Bcd molecules $c_{\text{clust}}$, and Bcd’s diffusion constant represented by *D*. The fractional error δN in counting *N* molecules within the cluster is given by

$$\frac{\partial N}{N}={\left(\frac{6}{D\cdot d\cdot c_{\text{clust}}\cdot T_{\text{clust}}}\right)}^{1/2}$$ (10)

Rearranging Eq. 10, we get

$$T_{\text{clust}}=\frac{6}{D\cdot d\cdot c_{\text{clust}}}{\left(\frac{\partial N}{N}\right)}^{-2}$$ (11)

The time $T_{\text{clust}}$ in Eqs. 10 and 11 represents the duration required for a cluster to “measure” the nuclear concentration with an accuracy of $\left(\frac{\partial N}{N}\right)$.

The analogous time for a binding site of length *a* to measure the nuclear concentration is given by

$$T_{\text{en}}=\frac{1}{D\cdot a\cdot c_{\text{nuc}}}{\left(\frac{\partial N}{N}\right)}^{-2}$$ (12)

Here, $c_{\text{nuc}}$ is the nuclear concentration. Using Eqs. 11 and 12 we derive the ratio

$$\frac{T_{\text{en}}}{T_{\text{clust}}}=\frac{d}{6a}\left(\frac{c_{\text{clust}}}{c_{\text{nuc}}}\right)$$ (13)

The length of a typical binding site can be considered as a=3.4 nm (*35*). However, *d*, $c_{\text{nuc}}$, and $c_{\text{clust}}$ depend on the nuclear position, thereby making $T_{\text{en}}/T_{\text{clust}}$ a position-dependent quantity.

### PSF measurements

To determine the PSF of the imaging system, 100-nm fluorescent polystyrene beads (Thermo Fisher Scientific catalog no. T14792) were imaged using the fast Airyscan mode on a Zeiss LSM 880 microscope. The objective is a 63× objective (Zeiss Plan-Apochromat 63×-1.4 oil immersion). Beads were illuminated with a 488-nm argon laser line. Images were acquired with a voxel size of 42 × 42 × 42 nm. A total thickness of 1 μm was imaged with the beads at the center. The beads are mounted on a flat glass surface and hence all reside on the same imaging plane. Each field of view contains 20 to 30 beads. Several such images were acquired. Pixel correlation was performed on these images, and the average correlation length provided the PSF of the system.

### Sequences for strong and weak enhancer constructs

WT variant:

CACGCTAGCTGCCTACTCCTGCTGTCGACTCCTGACC-AACGTAATCCCC

ATAGAAAACCGGTGGAAAATTCGCAGC-TCGCTGCTAAGCTGGCCATCCG

CTAAGCTCCCGGATCATC-CAAATCCAAGTGCGCATAATTTTTTGTTTCT

GCTCTAATC-CAGAATGGATCAAGAGCGCAATCCTCAATCCGCGATCC-GT

GATCCTCGATTCCCGACCGATCCGCGACCTGTACCT-GACTTCCCGTCAC

CTCTGCCCATCTAATCCCTTGACGCGT-GCATCCGTCTACCTGAGCGATA

TATAAACTAATGCCTGTT-GCAATTGTTCAGTCAGTCACGAGTTTGTTAC

CACTGCGA-CAACACAACAGAAGCAGCACCAATAATATACTTGCA-AATCC

TTACGAAAATCCCGACAAATTTGGAATATACTTC-GATACAATCGCAATC

ATACGCACTGAGCGGCCACGAAA-CGGTAGGA

All weak variant:

CACGCTAGCTGCCTACTCCTGCTGTCGACTCCTGACCAA-CGTAAGCTCC

ATAGAAAACCGGTGGAAAATTCGCAGCT-CGCTGCTAAGCTGGCCATCCG

CTAAGCTCCCGGATCATC-CAAATCCAAGTGCGCATAATTTTTTGTTTCT

GCTCTAAGC-TAGAATGGATCAAGAGCGCAATCCTCAATCCGCGATCC-GT

GATCCTCGATTCCCGACCGATCCGCGACCTGTACCT-GACTTCCCGTCAC

CTCTGCCCATCTAAGCTCTTGACGC-GTGCATCCGTCTACCTGAGCGATA

TATAAACTAATGCCT-GTTGCAATTGTTCAGTCAGTCACGAGTTTGTTAC

CACT-GCGACAACACAACAGAAGCAGCACCAATAATATACTTG-CAAATCC

TTACGAAAATCCCGACAAATTTGGAATATAC-TTCGATACAATCGCAATC

ATACGCACTGAGCGGCCACGA-AACGGTAGGA

All strong variant:

CACGCTAGCTGCCTACTCCTGCTGTCGACTCCTGAC-CAACGTAATCCCC

ATAGAAAACCGGTGGAAAATTCG-CAGCTCGCTGCTAATCCGGCCATCCG

CTAATCCCCCG-GATAATCCTAATCCAAGTGCGCATAATTTTTTGTTTCT-

GCTCTAATCCAGAATGGATTAAGAGCGTAATCCTTAATCC-GCGATCCGT

AATCCTCGATTCCCGACCGATCCGCGACCT-GTACCTGACTTCCCGTCAC

CTCTGCCCATCTAATCCCTT-GACGCGTGCATCCGTCTACCTGAGCGATA

TATAAACTAAT-GCCTGTTGCAATTGTTCAGTCAGTCACGAGTTTGTTAC-

CACTGCGACAACACAACAGAAGCAGCACCAATA-ATATACTTGCAAATCC

TTACGAAAATCCCGACAAATTTG-GAATATACTTCGATACAATCGCAATC

ATACGCACTGAGCG-GCCACGAAACGGTAGGA

## Supplementary Materials

### This PDF file includes:

Figs. S1 to S17 Table S1 References
